# Effects of Different Dietary Combinations on Blood Biochemical Indicators and Rumen Microbial Ecology in Wenshan Cattle

**DOI:** 10.3390/microorganisms12112154

**Published:** 2024-10-26

**Authors:** Dongwang Wu, Xiaoming He, Ying Lu, Zhendong Gao, Yuqing Chong, Jieyun Hong, Jiao Wu, Weidong Deng, Dongmei Xi

**Affiliations:** Yunnan Provincial Key Laboratory of Animal Nutrition and Feed Science, Faculty of Animal Science and Technology, Yunnan Agricultural University, Kunming 650201, China; danwey@163.com (D.W.); xiaominghe@foxmail.com (X.H.); yinglu_1998@163.com (Y.L.); zander_gao@163.com (Z.G.); 2022004@ynau.edu.cn (Y.C.); hongjieyun@163.com (J.H.); 15229238680@163.com (J.W.); dengwd@ynau.edu.cn (W.D.)

**Keywords:** Wenshan cattle, rumen microbiota, diet composition, intensive feeding, blood biochemical indicators

## Abstract

With the continuous optimization of feed ingredients in livestock production, barley has garnered significant attention as a potential substitute for corn in feed. This study aims to investigate the effects of replacing part of the corn and soybean meal with barley, wheat bran, and rapeseed meal on Wenshan cattle, focusing on the rumen microbial community, blood physiological and biochemical indicators, and growth traits. Through an intensive feeding experiment with two different dietary ratios, we found that adding barley to the diet significantly reduced the host’s blood lipid concentration and significantly increased the height, body length, heart girth, and average daily weight gain of Wenshan cattle. Analysis of the rumen microbial community structure showed that the addition of barley significantly affected the relative abundance of Firmicutes, Proteobacteria, and Bacteroidetes, with the relative abundance of Spirochaetes being significantly lower than that of the control group (*p* < 0.05). The dominant bacterial groups mainly included Acinetobacter, Solibacillus, and Lysinibacillus. In summary, this study reveals the potential of different feed ingredient ratios involving barley, wheat bran, and rapeseed meal in the production performance of Wenshan cattle. By regulating blood physiology and improving the rumen micro-ecological structure, it provides new scientific evidence for optimizing livestock and poultry feeding management strategies. Future research will further explore the optimal application ratio of barley under different feeding conditions and its long-term impact on animal health and production performance.

## 1. Introduction

With growing concerns about animal husbandry health, environmental protection, and sustainable development, optimizing feed ingredients has become a crucial strategy for improving livestock and poultry production performance and environmental sustainability [1,2,3]. Barley, as a potential alternative feed source, not only has nutritional advantages but may also significantly impact the host’s health and rumen microbiota [4]. The composition of feed directly influences the growth, development, and health status of animals, and also affects their digestion, absorption, immune function, and disease resistance by regulating the structure of the gut microbiota [5,6]. Therefore, optimizing feed formulations, particularly exploring the application of different feed ingredients, has become a key focus of current livestock and poultry production research [7]. In livestock feeding, corn and soybean meal have long been widely used as the main sources of energy and protein [8]. In addition to their nutritional benefits, the selection of dietary ingredients is significantly influenced by their availability and price. Ingredients such as barley, wheat bran, and rapeseed meal must be considered not only for their nutritional contributions but also for their local availability and cost-effectiveness. These practical aspects are crucial for formulating diets that are both nutritionally adequate and economically viable, ensuring that the proposed dietary interventions can be realistically implemented in diverse farming settings. Barley is a versatile feed ingredient known for its favorable nutritional profile. It offers several advantages in terms of price compared to corn. In many regions, barley is more cost-effective due to its lower production costs and higher local availability. This makes barley an attractive option for feed formulations, particularly in areas where corn prices are volatile. Wheat bran, a byproduct of wheat milling, provides high fiber content and contributes to digestive health. Its cost is generally lower than that of whole grains, making it an economical choice for improving digestive function and microbiota diversity in cattle. The availability of wheat bran is relatively stable, which adds to its cost-effectiveness as a feed ingredient. Rapeseed meal is a valuable protein source derived from the processing of rapeseed. It is often more affordable than soybean meal, especially in regions where rapeseed is a major crop. Rapeseed meal offers a good balance of protein and energy, making it a viable alternative to more expensive protein sources in feed formulations [3,9]. Barley, wheat bran, and rapeseed meal have drawn widespread interest as potential alternative ingredients due to their rich nutritional composition and environmentally friendly characteristics. In ruminant feed, barley, as a primary energy source, contains high levels of fiber and starch, which can significantly affect the structure and function of the rumen microbiota [6,10]. Studies have shown that adding barley can not only improve the growth performance of ruminants but also regulate their rumen fermentation characteristics and blood biochemical indicators [11]. Furthermore, wheat bran, a byproduct of barley processing, is rich in fiber and trace elements [12], significantly influencing the digestive function and diversity of the rumen microbiota in ruminants [13]. Rapeseed meal, a plant protein source rich in amino acids, can partially replace soybean meal in ruminant feed [14,15]. Research indicates that rapeseed meal not only provides essential nutrients but may also affect the structure and function of the rumen microbiota, thereby impacting the overall health and production performance of the animals [16]. Although these alternatives show potential in theory, their practical application still faces challenges. For instance, alternative ingredients may lead to changes in ruminants’ feed preferences and adaptability of the rumen microbiota. Therefore, a deeper understanding of the effects of these alternatives on ruminants can help optimize feed formulations and promote the sustainable development of ruminant farming systems. Specifically, whether the addition of barley during the fattening process can enhance weight gain, improve blood physiological indicators, and regulate the structure of the rumen microbiota requires further in-depth research and verification.

While the potential of barley as a feed ingredient has been widely discussed, its specific mechanisms of action and optimal application under different dietary combinations remain to be further explored. Therefore, this study aims to investigate the effects of replacing corn and soybean meal with barley and other feed ingredients on the physiological and biochemical indicators, rumen microbiota, and production performance of Wenshan cattle during intensive feeding, providing scientific evidence and theoretical support for optimizing dietary formulations.

## 2. Materials and Methods

### 2.1. Experimental Animals and Experimental Design

This experiment was conducted from 5 May 2022 to 28 October 2022, at the Guduonongmu Pasture in Wenshan Zhuang and Miao Autonomous Prefecture, Yunnan Province. A total of 20 healthy, well-nourished 30-month-old Wenshan cows were randomly selected and divided into two groups, with 10 cows in each group. The feeding trial lasted for 130 days, including a 10-day pre-feeding period and a 120-day formal feeding period. The daily fattening feed (control group diet), fattening feed ingredients (experimental group diet), and dry straw used in the experiment were all sourced from Wenshan, Yunnan, China, Guduonongmu Co., Ltd. The formulation and nutritional levels are detailed in Table 1. At the end of the feeding period, 50 mL of rumen fluid was collected via an oral tube, divided into 10 mL cryogenic tubes, and stored in liquid nitrogen for subsequent 16S rRNA sequencing. On the first morning of both the start and end of the feeding trial, the body weight, height, body length, chest girth, and heart girth of the Wenshan cows were measured using a measuring stick and tape measure, following the methods described in the “Cattle Production Science”. During measurement, the cows were allowed to stand naturally on a flat surface. The statistical data for body traits are presented as “mean value ± standard deviation”.

Blood Sample Collection: On the morning of the first day after the feeding period ended, blood samples were collected using the tail vein method into 5 mL heparinized vacuum blood collection tubes. The tubes were then tilted and left to stand for 30 min. After standing, the tubes were placed in a centrifuge, set to 3000 r/min, and centrifuged for 15 min. The supernatant was aliquoted into 1.5 mL centrifuge tubes using a pipette and stored in a −20 °C freezer for subsequent blood biochemical analysis. Plasma biochemical indicators were measured using an automated blood analyzer (Roche physiological biochemistry instrument(Basel, Switzerland), including glutamyl transpeptidase (GGT), alanine aminotransferase (ALT), alkaline phosphatase (ALP), aspartate transaminase (AST), serum albumin (ALB), globulin (GLOB), total protein (TP), indirect bilirubin (IBIL), direct bilirubin (DBIL), total bilirubin (TBIL), triglyceride (TG), low-density lipoprotein cholesterol (LDL-CH), high-density lipoprotein cholesterol (HDL-CH), cholesterol (CHOL), urea (UREA), and glucose (GLU).

### 2.2. Sequencing and Data Analysis

The rumen fluid samples collected from the 20 Wenshan cows were sent to Guangzhou Gideo Biological Technology Co., Ltd. (Guangzhou, China). for the following experimental procedures. Bacterial genomic DNA was extracted from the rumen fluid according to the requirements of the kit, and the concentration and purity were detected using a NanoDrop micro-spectrophotometer (OD value 260/280) (Thermo Fisher Scientific, Waltham, MA, USA). The integrity of the DNA was checked using 1% agarose gel electrophoresis. The bacterial 16S rDNA V3~V4 region was amplified using universal primers 341F (5-CCTACGGGNGGCWGCAG-3) and 806R (5-GGACTACHVGGGTATCTAAT-3). The qualified DNA samples were subjected to sequence amplification, and the PCR amplification products were recovered from the gel and quantified using a QuantiFluorTM fluorometer (Promega, Madison, WI, USA). The purified amplification products were mixed in equal amounts, linked to sequencing adapters, and used to construct sequencing libraries for subsequent sequencing.

After obtaining the raw reads from sequencing, low-quality reads were filtered out, and the remaining reads were assembled, with paired-end reads being merged into tags. These tags were further filtered to produce clean tags. Next, clean tags were clustered, and any chimeric tags detected during clustering were removed, yielding effective tags. After obtaining operational taxonomic units (OTUs), OTU abundance was calculated based on the effective tags. Representative sequences were selected during the OTU/ASV construction process, and these sequences were annotated using the naïve Bayesian assignment algorithm of the RDP classifier against a database. The top 10 species with the highest mean abundance across all samples were detailed, with other species categorized under “Other,” and unannotated tags classified as “Unclassified.” Boxplots were drawn based on Tukey HSD test results. Differential group analysis was performed using LEfSe software (Version 1.0). PCoA plots were generated using the R language’s ggplot2 package, and alpha diversity analysis was conducted using Qiime. Circos plots were drawn to depict species distribution, and Pearson correlation coefficients were calculated based on species abundance tables using the psych package in R (Version 3.5.3). Statistical significance was assessed using Fisher’s Z-transformation, and relationships with a correlation coefficient greater than 0.5 were used to construct network diagrams using igraph (Version 1.6.0).

## 3. Results

### 3.1. Growth Performance of Wenshan Cattle

Various dietary compositions induced certain alterations in the body weight and size of Wenshan cattle; however, these differences did not reach statistical significance, as evidenced by the data presented in Table 2. Both groups exhibited an augmentation in body size index traits, with the experimental group demonstrating a notably greater increase in both body weight and body size traits compared to the control group. Notably, there was no substantial distinction in tube circumference between the control and experimental groups before the initiation of the experiment (*p* > 0.05). Nevertheless, following the experiment, the tube circumference of the experimental group exhibited a significant increase compared to that of the control group (*p* < 0.05).

### 3.2. Blood Index of Wenshan Cattle

After a brief period of fattening, the blood liver function indices in Wenshan cattle remained within the normal range of physiological parameters, with the exception of ALP, which was lower than that observed in normal cattle (refer to Table 3). In comparison with the control group, the experimental groups exhibited a significant increase in ALT, IBIL, DBIL, and TBIL levels (*p* < 0.01), while the AST/ALT ratio experienced a significant decrease (*p* < 0.05). Other liver function indices showed no significant differences (*p* > 0.05). Among the four lipid indices, only cholesterol surpassed the normal range value for cattle, while the remaining parameters fell within the normal range. Notably, the experimental groups demonstrated a significant reduction in LDL-CH and CHOL levels in blood lipid indices (*p* < 0.01), along with a significant decrease in HDL-CH levels (*p* < 0.05), while TG levels showed no significant difference (*p* > 0.05) compared to the control group. The blood urea content in Wenshan cattle after the short-term fattening period exhibited a significant increase compared to that of the control group (*p* < 0.01). Both groups displayed blood glucose content exceeding the normal range value, with no significant differences observed between the two experimental groups and the control group (*p* > 0.05).

### 3.3. Rumen Microorganism

#### 3.3.1. Quality Control of Rumen Microbial Original Sequencing Data

**Figure 1:**
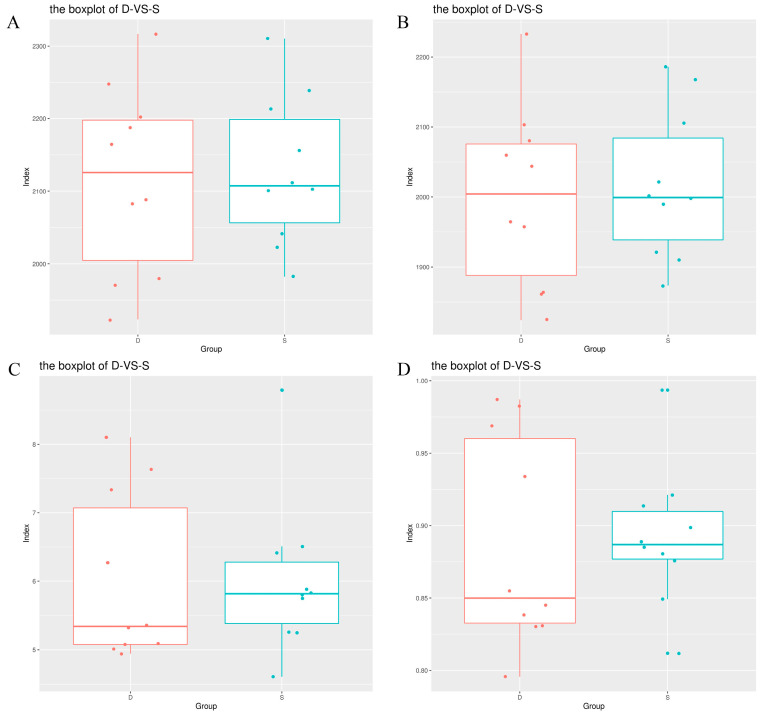
The Tukey HSD test’s box plot is depicted in the diagram. The horizontal axis signifies the grouping, while the vertical axis represents the extent of the diversity index. The midpoint symbolizes the sample. The labels correspond to specific diversity indices: (**A**) (Ace), (**B**) (Chao1), (**C**) (Shannon), and (**D**) (Simpson).

Following gene sequencing utilizing 16S rDNA technology, a total of 2,619,567 pairs of reads were obtained from 20 samples, encompassing both the test and control groups. The paired reads were subsequently spliced and filtered, resulting in 2,531,055 clean tags (representing the number of high-quality tags obtained after quality control). Each sample generated a minimum of 116,445 clean tags, with an average of 126,553 clean tags. Through clustering based on clean tags and the removal of chimeric tags detected during the clustering process, a total of 223,485 effective tags (indicating the number of high-quality tags after the exclusion of chimeras, i.e., tags suitable for subsequent analysis) were obtained, constituting 87.04% of the original data. The tags, clustered and annotated with a 97% similarity level, yielded an average of 1883 operational taxonomic units (OTUs) for each sample. Alpha diversity index analysis revealed that the richness indices of rumen flora, including Chao1, Ace index, Shannon diversity index, and Simpson index, were all higher in the experimental groups compared to the control group, though without reaching statistical significance (*p* > 0.05) (Figure 1).

**Figure 2:**
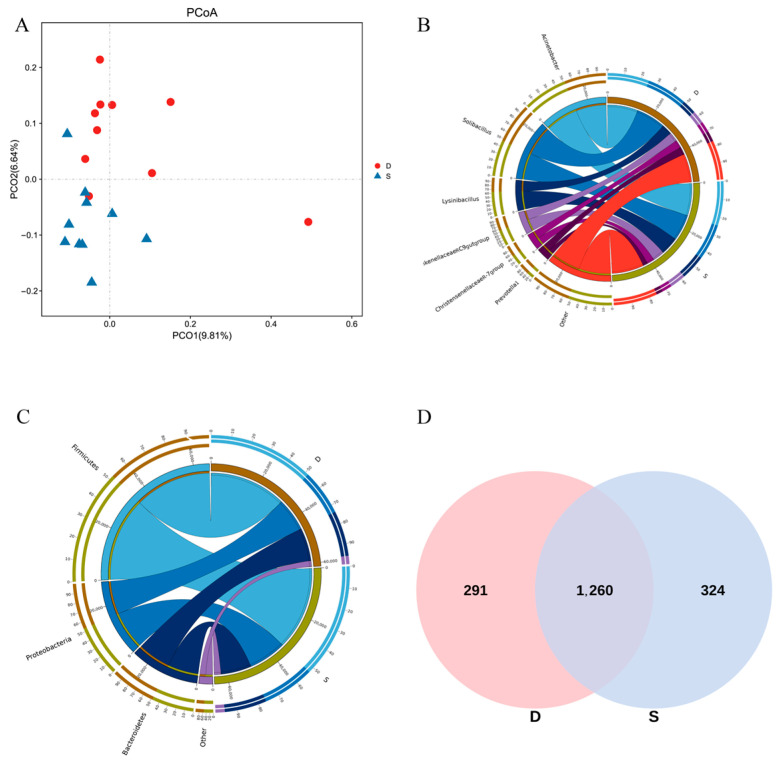
(**A**) PCoA scatter plot based on inter-sample Beta diversity index. (**B**) Group phylum level circos graph. (**C**) Group genus horizontal circos graph. (**D**) The graph of OTU venn.

In Beta diversity analysis, the unweighted UniFrac method was employed for principal coordinates analysis (PCoA), as illustrated in Figure 2A. PCoA scatter plots reveal distinctions between individuals or groups. The first principal component (PCo1) explained 9.81% of the variation, while the second principal component (PCo2) explained 6.64%. Despite the close proximity and clustered distribution observed between the experimental and control groups, indicating a small difference in microbial communities, the community similarity remained high.

#### 3.3.2. Analysis of Species Abundance Composition

The Circos diagram at the phylum level (Figure 2B) illustrates the top three species with relative abundance. *Firmicutes*, *Proteobacteria*, and *Bacteroidetes* were identified as the primary connections between the control and experimental groups. These findings underscored *Firmicutes*, *Proteobacteria*, and *Bacteroidetes* as the predominant bacterial phyla in both groups. As presented in Table 4, the collective relative abundance of these three dominant bacterial phyla in the control and experimental groups was 95.013% and 94.998%, respectively. Although the experimental group exhibited a marginal increase in the relative abundance of *Firmicutes* and *Proteobacteria* by 2.367% and 2.208%, respectively, in comparison to the control group, these differences were not statistically significant (*p* > 0.05). Conversely, the relative abundance of *Bacteroidetes* in the experimental group decreased by 3.87%, yet this difference was not statistically significant (*p* > 0.05). The relative abundance of other bacterial phyla was generally lower, with *Spirochaetes* displaying a significantly lower abundance in the experimental group compared to the control group (*p* < 0.05).

The comparative distribution of rumen bacteria at the genus level in both the control and experimental groups is illustrated in Figure 2C. The predominant genera identified in both groups include *Acinetobacter*, *Solibacillus*, and *Lysinibacillus*. As outlined in Table 5, the relative abundance of these three bacterial genera in the control and experimental groups was 47.417% and 50.585%, respectively. Notably, the experimental group exhibited a slightly higher relative abundance of these dominant genera compared to the control group, although this difference did not reach statistical significance (*p* > 0.05). A Venn diagram (Figure 2D) reveals a total of 1875 operational taxonomic units (OTUs) within the rumen of Wenshan cattle across both groups, with 1260 OTUs shared between them. Group D and Group S exhibited 291 and 324 unique species, respectively. In summary, the aggregate count of total and unique species is notably higher in Group S.

#### 3.3.3. Analysis of Differential Microbial Composition

**Figure 3:**
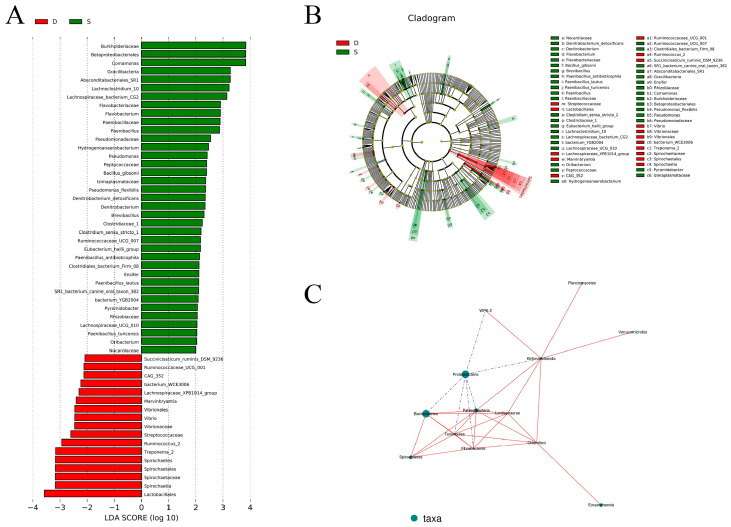
(**A**) LEFse difference analysis graph. (**B**) Cladogram. In an evolutionary cladistic diagram, the circles radiating from the inside out represent taxonomic levels from phylum to genus (or species). Each small circle at a different classification level represents a classification at that level, and the size of the small circle diameter is proportional to the relative abundance size. Coloration principle, species with no significant differences are uniformly colored yellow. (**C**) Species correlation network graph at the phylum level.

In examining specific microorganisms within the control and experimental groups, we employed LEfSe, setting the LAD threshold to exceed 3.0, as depicted in Figure 3A. Notably, the experimental group exhibited enrichment in *Burkholderiaceae*, *Betaproteobacteriales*, *Comamonas*, *Gracilibacteria*, and Abscond group SR1, with predominant taxa including *Itabacteriales_sR1*, *Lachnoclostridium_10*, and *Lachnospiraceae_bacterium_cG2*. Conversely, the control group featured enrichment in Toxoplasma type II (*Treponema_2*), *Spirochaetes*, *Spirochaetales*, *Spirochaetaceae*, *Spirochaetia*, and *Acidophilus* (La), particularly on *Clostridiales*. Notably, the highest LAD value was observed in *Betaproteobacteriales*.

#### 3.3.4. Analysis of Species Correlation

We constructed an association network to examine correlations among bacterial expression levels characterized by higher abundance (Figure 3C). Each node within the network represents a distinct dominant genus, and the connections between the nodes signify correlations between the respective entities. The strength of the correlation is visually depicted by connection lines, with red lines denoting positive correlations and blue lines indicating negative correlations. The number of node connections facilitates the identification of species with closer relationships within the microbial community. In this investigation, *Proteobacteria*, exhibiting high abundance, demonstrated a noteworthy negative correlation with *Bacteroidetes*. Furthermore, *Bacteroidetes* displayed correlations with *Spirochaetes*, *Fibrobacteres*, *Lentisphaerae*, and *Patescibacteria*.

## 4. Discussion

This study explored the effects of different dietary combinations under intensive feeding on the production performance, blood biochemical indicators, and rumen microbiota of Wenshan cattle. The optimized diet combination for the experimental group indicated that barley, wheat bran, and rapeseed meal can partially replace corn and soybean meal while improving the average daily weight gain of Wenshan cattle. These results are consistent with previous studies, which have shown that intensive feeding can enhance the growth performance of ruminants, improve rumen fermentation characteristics, and modify microbial communities and blood biochemical parameters [17,18]. The observed changes in body weight and blood biochemical indicators highlight the effectiveness of the intensive feeding regimen employed in this study.

The blood biochemical markers measured in this study provide valuable insights into the metabolic status and health of the animals under different dietary interventions. Serum biochemical indicators reflect metabolic changes in animal tissues and organs [19]. Serum transaminases, total bilirubin, and LDL-CH directly indicate the metabolic status of the liver. Some studies have shown that blood bilirubin levels are negatively correlated with liver fat accumulation [20]. Dietary changes can influence animal feed intake and alter individual blood lipid levels [21,22]. The analysis of blood biochemical parameters in this study revealed significant changes in liver function markers and blood lipid profiles in Wenshan cattle under different dietary combinations of intensive feeding. Elevated levels of ALT, IBIL, DBIL, and TBIL in the experimental group suggest possible liver stress or adaptation to the high-energy diet. Interestingly, the decrease in HDL-CH and LDL-CH levels in the experimental group contrasts with findings from traditional intensive feeding studies, indicating a significant metabolic response difference to the specific dietary combination used.

Glucose levels, for instance, serve as a critical indicator of energy metabolism. In our study, we observed a reduction in glucose levels in animals receiving a high-fiber diet, which suggests an improvement in the regulation of blood sugar due to better carbohydrate utilization. This aligns with findings from previous studies where high-fiber diets promoted stable glucose levels by slowing the rate of carbohydrate absorption in the gut [23]. Lower glucose levels might indicate better control of blood sugar and reduced the risk of metabolic disorders, suggesting that the diet had a positive impact on energy metabolism. Additionally, triglyceride concentrations showed a decrease in animals on the fiber-rich diet. This reduction suggests that the high-fiber content influenced lipid metabolism, potentially enhancing fat mobilization and reducing fat storage. Similar findings have been reported in other studies where dietary fiber enhanced lipid metabolism by reducing fat absorption and increasing fat excretion [24]. This metabolic adjustment is critical for maintaining energy balance, particularly in ruminants, where efficient fat metabolism is vital for milk production and growth performance.

The composition of the rumen microbiota plays a critical role in digestion and overall health in ruminants, as it is responsible for breaking down complex plant fibers and producing volatile fatty acids (VFAs), which are the primary energy source for these animals. In our study, we observed significant shifts in the microbial community structure in response to diet changes, particularly in fiber-degrading bacteria such as the *Ruminococcus* genus. An increase in *Ruminococcus* species suggests an enhanced ability to degrade plant fibers, which is essential for maximizing nutrient extraction from fibrous diets. This shift is crucial because improved fiber digestion can lead to more efficient feed utilization, translating to better growth performance and productivity in livestock [25]. The rumen microbiota plays a crucial role in the host’s nutrient metabolism, product quality, and environmental impact [26,27]. There is a close relationship between rumen microbial communities and host production performance [28,29,30]. Studies have shown that different dietary compositions, ingredients, and intensive feeding methods significantly regulate rumen microbial structure and fermentation levels [31,32,33]. Conversely, we observed a reduction in the relative abundance of *Prevotella*, a genus involved in the fermentation of carbohydrates and protein. The high-fiber, low-starch diet may have suppressed the proliferation of these carbohydrate-fermenting bacteria, leading to a shift towards fiber fermentation. This is important because changes in microbial composition can alter the rumen fermentation profile, specifically the types and amounts of VFAs produced. Increased production of acetate, for instance, is often associated with high-fiber diets and is critical for milk fat synthesis in dairy cattle [34]. This shift towards more acetate production could improve fat metabolism, contributing to healthier animals and better milk production. The shifts in the microbial community structure also have implications for animal health. For example, a more diverse and stable microbiota, enriched in fiber-degrading bacteria, can improve gut health and reduce the risk of rumen acidosis, a common issue in ruminants fed high-starch diets [35,36]. By promoting the growth of fiber-degrading bacteria, the high-fiber diet may have supported a more stable rumen environment, which is essential for maintaining optimal digestive function and preventing metabolic disorders.

Additionally, the observed increase in *Butyrivibrio* species, known for their role in butyrate production, is another positive indicator of gut health. Butyrate is a key VFA that promotes the health of rumen epithelial cells and improves nutrient absorption [37,38]. Increased butyrate production may contribute to enhanced digestive efficiency and overall animal health, further supporting the benefits of the high-fiber diet.

The dominance of *Firmicutes*, *Proteobacteria*, and *Bacteroidetes* in both groups indicates that, despite dietary interventions, the rumen microbiota composition remained relatively stable. Notably, the relative abundance of *Spirochaetes* in the experimental group was significantly lower than in the control group, although there were no significant differences in other phyla. *Spirochaetes* have been shown to play a vital role in feed digestion and short-chain fatty acid production in the animal gut [39]. At the genus level, the dominant bacterial genera in both the control and experimental groups were *Acinetobacter*, *Solibacillus*, and *Lysinibacillus*. *Solibacillus* and *Lysinibacillus* are known to promote feed fermentation [40], suggesting that the combination of barley, wheat bran, rapeseed meal, and corn in the diet may also favor rumen fermentation. In summary, the changes in the rumen microbiota in response to dietary intervention suggest a shift towards a more fiber-centric microbial community. This shift not only improves fiber digestion and fermentation but also enhances overall gut health and nutrient absorption, which could lead to better performance outcomes in livestock.

Generalization to ther Breeds or Species. This study focused on Wenshan cattle, a local breed in the highland areas of southern China with unique physiological traits and adaptations. However, we believe that some findings from this study may have broader applicability, especially among ruminants. Despite differences in genetic traits among cattle breeds, which might lead to varying physiological responses to dietary changes, research has shown that the impact of fiber-based diets on rumen microbiota structure and metabolic health exhibits similar mechanisms across different ruminants [41,42]. The effect of fiber on the rumen microbial community structure and fermentation processes is relatively consistent among ruminants. Therefore, the insights gained from this study regarding the impact of fiber-based diets on blood biochemical markers, rumen microbiota structure, and metabolic functions may be applicable to other cattle breeds and ruminant species. However, given the genetic and environmental differences, further research across different breeds and under varying feeding conditions is needed to validate the broader applicability of these results.

Impact of Environmental Factors. We also recognize that environmental factors such as climate, resource availability, and management practices could significantly influence the generalizability of our findings. For instance, in tropical or arid regions, where forage quality is lower and heat stress is more prevalent, the response of cattle to fiber-based diets might differ [43]. In such climatic conditions, the feed conversion efficiency and stability of the rumen microbiota could be challenged, potentially leading to different outcomes in blood biochemical markers and metabolic health. Additionally, the availability of feed resources, including the type and quality of forages and roughages, might impact the effectiveness of fiber-based diets on animal health and production performance. Therefore, to better understand the practical implications of dietary adjustments, further studies in various climatic conditions and resource environments are recommended to ensure the general applicability of these dietary strategies.

## 5. Conclusions

This study revealed that different dietary combinations can significantly improve the health and production performance of Wenshan cattle. Adding barley, wheat bran, and rapeseed meal to the diet alters rumen microbiota diversity, creating a more balanced microbial community. This enhancement supports more effective feed digestion and nutrient absorption, ultimately boosting growth performance and reducing metabolic disorders. Incorporating these dietary components is an effective strategy for optimizing feed efficiency and overall animal health. This approach provides valuable insights for nutritionists and farmers seeking to enhance livestock productivity and sustainability.

For future research, we recommend the following: (1) Cross-Breed and Species Studies: Investigate whether the observed benefits extend to other cattle breeds and ruminant species to assess the broader applicability of our findings; (2) Long-Term Effects and Economic Analysis: Conduct long-term studies to evaluate the sustained impact of these dietary interventions on cattle health, productivity, and economic viability, including a cost-effectiveness analysis; (3) Environmental and Climatic Variations: Explore how environmental factors, such as climate and forage availability, influence the effectiveness of these dietary components to tailor feeding strategies to different conditions; and (4) Microbiota and Metabolic Interactions: Study the specific interactions between rumen microbiota and dietary components to understand their effects on metabolic processes and overall health, leading to more targeted nutritional strategies. Addressing these areas will provide deeper insights and more comprehensive guidelines for optimizing cattle nutrition and management.

## Figures and Tables

**Table 1:** Nutrient level and composition of experimental diets (on a dry matter basis).

Ingredients	Component Ratio (%)	
	Control Group	Experimental Group
Corn	60	30
Soybean meal	12	5
Rapeseed meal	0	6
barley	0	40
White sugar	0	2
Wheat bran	0	10
Calcium hydrogen phosphate	1	0.75
salt	1	0.5
Sodium carbonate	1	0.75
additive	0	2
Vegetable oil	0	3
Concentrate feed	15	0
molasses	10	0
total	100	100
Digestible energy DE (MJ/Kg)	13.73	13.55
Crude protein (CP)	16.22	13.12
Ether extract (EE)	2.4	2.33
Neutral detergent fibre (NDF)	7.21	15.77
Acid detergent fibre (ADF)	2.77	6.32
Calcium (Ca)	0.21	0.29
phosphorus (P)	0.31	0.4

Note: The premix contains the following nutrients per kilogram: Vitamin A 200,000 IU, Vitamin D 340,000 IU, Vitamin E 600 mg, Niacin 400 mg, Manganese 700 mg, Zinc 2000 mg, Iron 800 mg, Copper 500 mg, Iodine 6 mg, Selenium 25 mg, and Cobalt 5 mg.

**Table 2:** Difference in body weight and body measurement traits of Wenshan cattle before and after the experiment.

Index	Item	Control Group	Experimental Group
Body weight (Kg)	Before the experiment	188.86 ± 56.69	203.64 ± 12.95
	After the experiment	293.71 ± 52.03	311.57 ± 30.44
	Gain weight	104.86 ± 14.31	107.92 ± 29.96
	Average daily gain	0.8729 ± 0.12	0.90 ± 0.25
Body height (cm)	Before the experiment	116 ± 3.05	117.86 ± 3.13
	After the experiment	118.43 ± 3.55	121.00 ± 2.77
	Gain	2.43 ± 1.62	3.14 ± 1.95
	Average growth	0.02 ± 0.01	0.03 ± 0.02
Body length (cm)	Before the experiment	117.86 ± 9.79	123.14 ± 4.63
	After the experiment	125.86 ± 8.93	133.85 ± 11.28
	Gain	8.00 ± 7.90	10.71 ± 9.27
	Average growth	0.07 ± 0.07	0.09 ± 0.08
Chest circumference (cm)	Before the experiment	141.00 ± 13.51	149.71 ± 5.40
	After the experiment	168.57 ± 7.63	170.00 ± 4.16
	Gain	22.57 ± 8.94	20.29 ± 4.42
	Average growth	0.02 ± 0.008	0.02 ± 0.01
Canno round (cm)	Before the experiment	15.29 ± 1.80	14.57 ± 0.98
	After the experiment	14.43 ± 0.53	15.14 ± 0.38 *
	Gain	0.86 ± 1.57	0.57 ± 0.98
	Average growth	0.009 ± 0.01	0.006 ± 0.01
Dry matter(Kg)	Intake	5.13 ± 0.15	5.27 ± 0.18

Note: The peer data shoulder mark * in the table indicates a significant difference (*p* < 0.05), no labeling indicates that the difference was not significant, the same applies to the following tables.

**Table 3:** Blood biochemical indexes of Wenshan cattle.

Index	Control Group	Experimental Group	Normal Range
Glutamyl transpeptidase (GGT) (U/L)	25.36 ± 21.17	16.51 ± 3.84	/
Alanine aminotransferase (ALT) (U/L)	28.08 ± 6.02	35.98 ± 5.20 **	15–70
Alkaline phosphatase (ALP) (U/L)	6.98 ± 6.33	4.06 ± 2.96	18–153
Aspartate transaminase (AST) (U/L)	78.22 ± 19.52	76.81 ± 14.01	45–110
Aspartate transaminase/Alanine aminotransferase(S/L) (Ratio)	2.82 ± 0.68	2.16 ± 0.39 *	0.8–1.2
Serum albumin (ALB) (g/L)	36.58 ± 1.51	37.78 ± 3.13	21–36
Globulin (GLOB) (g/L)	40.79 ± 5.25	43.09 ± 8.56	34–48.5
Total protein (TP) (g/L)	77.37 ± 5.53	80.87 ± 6.40	57–81
Indirect bilirubin (IBIL) (μmol)	1.56 ± 0.64	2.58 ± 0.57 **	3–17
Direct bilirubin (DBIL) (μmol/L)	1.35 ± 0.41	1.99 ± 0.43 **	0.7–6.8
total bilirubin (TBIL) (μmol/L)	2.92 ± 0.85	4.57 ± 0.95 **	0.17–17.1
Triglyceride (TG) (mmol/L)	0.37 ± 0.32	0.29 ± 0.10	0.23–0.57
Low-density lipoprotein cholesterol (LDL-CH) (mmol/L)	1.64 ± 0.64	0.97 ± 0.20 **	0.65–1.81
High-density lipoprotein cholesterol (HDL-CH) (mmol/L)	2.43 ± 0.37	2.02 ± 0.36 *	0.4–2.6
CholesterolCHOL) (mmol/L)	4.91 ± 1.51	3.33 ± 0.47 **	3.1–5.69
Urea (UREA) (mmol/L)	3.95 ± 0.63	5.38 ± 0.73 **	2.8–7.5
Glucose (GLU) (mmol/L)	7.30 ± 4.14	6.76 ± 2.28	3.9–5.5

Note: The peer data shoulder mark * in the table indicates a significant difference (*p* < 0.05), Shoulder mark ** indicates a significant difference (*p* < 0.01).

**Table 4:** Relative abundance of rumen microbiota at phylum level in Wenshan cattle.

Item	Group		*p*
	Control	Experimental	
*Firmicutes*	50.913 ± 10.753	52.560 ± 8.941	0.703
*Proteobacteria*	20.893 ± 12.835	23.101 ± 13.152	0.709
*Bacteroidetes*	23.207 ± 12.541	19.337 ± 7.692	0.416
*Patescibacteria*	1.201 ± 0.556	1.345 ± 1.246	0.743
*Spirochaetes*	1.004 ± 0.399	0.671 ± 0.201 *	0.030
*Euryarchaeota*	0.705 ± 1.007	0.824 ± 0.823	0.776
*Actinobacteria*	0.445 ± 0.583	0.455 ± 0.338	0.963
*Tenericutes*	0.167 ± 0.084	0.230 ± 0.080	0.106
*Kiritimatiellaeota*	0.273 ± 0.158	0.331 ± 0.227	0.511
*Planctomycetes*	0.758 ± 0.290	0.692 ± 0.165	0.538

Note: The peer data shoulder mark * in the table indicates a significant difference (*p* < 0.05).

**Table 5:** Relative abundance of rumen microbiota at genus level in Wenshan cattle.

Item	Group		*p*
	Control	Experimental	
*Acinetobacter*	20.393 ± 12.953	20.940 ± 13.636	0.928
*Solibacillus*	19.993 ± 17.095	17.505 ± 13.675	0.724
*Lysinibacillus*	7.031 ± 6.575	12.135 ± 9.245	0.172
*Rikenellaceae_RC9_gut_group*	6.690 ± 1.963	7.794 ± 2.696	0.309
*Christensenellaceae_R-7_group*	5.719 ± 1.778	4.943 ± 1.025	0.248
*Prevotella_1*	5.859 ± 4.826	3.163 ± 1.310	0.105
*Succiniclasticum*	2.602 ± 1.960	1.731 ± 0.964	0.223
*Saccharofermentans*	1.660 ± 0.688	1.957 ± 0.472	0.275
*Ruminococcaceae_NK4A214_group*	1.497 ± 0.678	1.658 ± 1.069	0.693
*Candidatus_Saccharimonas*	1.014 ± 0.559	0.837 ± 0.672	0.530

## Data Availability

The datasets presented in this study can be found in online repositories. Bacterial and fungal sequences from this project are deposited in the National Center for Biotechnology Information (NCBI) Short Read Archive (SRA) under the BioProject number PRJNA1053428.

## References

[1] Groot M.J., Van’t Hooft K.E. (2016). The Hidden Effects of Dairy Farming on Public and Environmental Health in the Netherlands, India, Ethiopia, and Uganda, Considering the Use of Antibiotics and Other Agro-chemicals. Front. Public Health.

[2] Goni I., Brenes A., Centeno C., Viveros A., Saura-Calixto F., Rebole A., Arija I., Estevez R. (2007). Effect of dietary grape pomace and vitamin E on growth performance, nutrient digestibility, and susceptibility to meat lipid oxidation in chickens. Poult. Sci..

[3] Alimi N., Assani A.S., Sanni Worogo H., Baco N.M., Traore I.A. (2024). Livestock feed resources used as alternatives during feed shortages and their impact on the environment and ruminant performance in West Africa: A systematic review. Front. Vet. Sci..

[4] Winkler L.R., Hasenbeck A., Murphy K.M., Hermes J.C. (2017). Replacing Corn and Wheat in Layer Diets with Hulless Oats Shows Effects on Sensory Properties and Yolk Quality of Eggs. Front. Nutr..

[5] Huang Y., Jonsson N.N., McLaughlin M., Burchmore R., Johnson P.C.D., Jones R.O., McGill S., Brady N., Weidt S., Eckersall P.D. (2023). Quantitative TMT-based proteomics revealing host, dietary and microbial proteins in bovine faeces including barley serpin Z4, a prominent component in the head of beer. J. Proteom..

[6] Ma X., Zhou W., Guo T., Li F., Li F., Ran T., Zhang Z., Guo L. (2021). Effects of Dietary Barley Starch Contents on the Performance, Nutrient Digestion, Rumen Fermentation, and Bacterial Community of Fattening Hu Sheep. Front. Nutr..

[7] Li Y., Han Y., Zhao Q., Tang C., Zhang J., Qin Y. (2022). Fermented Soy and Fish Protein Dietary Sources Shape Ileal and Colonic Microbiota, Improving Nutrient Digestibility and Host Health in a Piglet Model. Front. Microbiol..

[8] He Z., Liu S., Wen X., Cao S., Zhan X., Hou L., Li Y., Chen S., Zheng H., Deng D. (2024). Effect of mixed meal replacement of soybean meal on growth performance, nutrient apparent digestibility, and gut microbiota of finishing pigs. Front. Vet. Sci..

[9] Maxin G., Ouellet D.R., Lapierre H. (2013). Effect of substitution of soybean meal by canola meal or distillers grains in dairy rations on amino acid and glucose availability. J. Dairy Sci..

[10] Zhang Z., Li F., Ma X., Li F., Wang Z. (2022). Effects of Barley Starch Level in Diet on Fermentation and Microflora in Rumen of Hu Sheep. Animals.

[11] Silveira C., Oba M., Yang W.Z., Beauchemin K.A. (2007). Selection of barley grain affects ruminal fermentation, starch digestibility, and productivity of lactating dairy cows. J. Dairy Sci..

[12] Chen M., Liu S., Imam K., Sun L., Wang Y., Gu T., Wen B., Xin F. (2020). The Effect of Xylooligosaccharide, Xylan, and Whole Wheat Bran on the Human Gut Bacteria. Front. Microbiol..

[13] Jiang X., Xu H.J., Ma G.M., Sun Y.K., Li Y., Zhang Y.G. (2021). Digestibility, lactation performance, plasma metabolites, ruminal fermentation, and bacterial communities in Holstein cows fed a fermented corn gluten-wheat bran mixture as a substitute for soybean meal. J. Dairy Sci..

[14] Rehemujiang H., Yusuf H.A., Ma T., Diao Q., Kong L., Kang L., Tu Y. (2023). Fermented cottonseed and rapeseed meals outperform soybean meal in improving performance, rumen fermentation, and bacterial composition in Hu sheep. Front. Microbiol..

[15] Goiri I., Zubiria I., Lavin J.L., Benhissi H., Atxaerandio R., Ruiz R., Mandaluniz N., Garcia-Rodriguez A. (2021). Evaluating the Inclusion of Cold-Pressed Rapeseed Cake in the Concentrate for Dairy Cows upon Ruminal Biohydrogenation Process, Ruminal Microbial Community and Milk Production and Acceptability. Animals.

[16] Agwa H.M.M., Saleh H.M., Ayyat M.S., Abdel-Rahman G.A. (2023). Effect of replacing cottonseed meal with canola meal on growth performance, blood metabolites, thyroid function, and ruminal parameters of growing lambs. Trop. Anim. Health Prod..

[17] Li B., Sun X., Huo Q., Zhang G., Wu T., You P., He Y., Tian W., Li R., Li C. (2021). Pelleting of a Total Mixed Ration Affects Growth Performance of Fattening Lambs. Front. Vet. Sci..

[18] Soltani E., Naserian A.A., Khan M.A., Ghaffari M.H., Malekkhahi M. (2020). Effects of conditioner retention time during pelleting of starter feed on nutrient digestibility, ruminal fermentation, blood metabolites, and performance of Holstein female dairy calves. J. Dairy Sci..

[19] Chen Y., Gong X., Li G., Lin M., Huo Y., Li S., Zhao G. (2016). Effects of dietary alfalfa flavonoids extraction on growth performance, organ development and blood biochemical indexes of Yangzhou geese aged from 28 to 70 days. Anim. Nutr..

[20] Hinds T.D., Creeden J.F., Gordon D.M., Stec D.F., Donald M.C., Stec D.E. (2020). Bilirubin Nanoparticles Reduce Diet-Induced Hepatic Steatosis, Improve Fat Utilization, and Increase Plasma beta-Hydroxybutyrate. Front. Pharmacol..

[21] Reis L.G., Silva T.H.D., Salles M.S.V., Andrade A.F.C., Martins S., Takeuchi P.L., Vidal A.M.C., Netto A.S. (2022). Effect of cow’s milk with different PUFA n-6: N-3 ratios on performance, serum lipid profile, and blood parameters of grower gilts. PLoS ONE.

[22] Noruzi S., Torki M., Mohammadi H. (2022). Effects of supplementing diet with Thyme (*Thymuas vulgaris* L.) essential oil and/or selenium yeast on production performance and blood variables of broiler chickens. Vet. Med. Sci..

[23] Muller M., Canfora E.E., Blaak E.E. (2018). Gastrointestinal Transit Time, Glucose Homeostasis and Metabolic Health: Modulation by Dietary Fibers. Nutrients.

[24] Zebeli Q., Aschenbach J.R., Tafaj M., Boguhn J., Ametaj B.N., Drochner W. (2012). Invited review: Role of physically effective fiber and estimation of dietary fiber adequacy in high-producing dairy cattle. J. Dairy Sci..

[25] Krause D.O., Denman S.E., Mackie R.I., Morrison M., Rae A.L., Attwood G.T., McSweeney C.S. (2003). Opportunities to improve fiber degradation in the rumen: Microbiology, ecology, and genomics. FEMS Microbiol. Rev..

[26] Xu Q., Ungerfeld E.M., Morgavi D.P., Waters S.M., Liu J., Du W., Zhao S. (2023). Editorial: Rumen microbiome: Interacting with host genetics, dietary nutrients metabolism, animal production, and environment. Front. Microbiol..

[27] Chen P., Li Y., Wang M., Shen Y., Liu M., Xu H., Ma N., Cao Y., Li Q., Abdelsattar M.M. (2024). Optimizing dietary rumen-degradable starch to rumen-degradable protein ratio improves lactation performance and nitrogen utilization efficiency in mid-lactating Holstein dairy cows. Front. Vet. Sci..

[28] Zhao X., Zhang Y., Rahman A., Chen M., Li N., Wu T., Qi Y., Zheng N., Zhao S., Wang J. (2024). Rumen microbiota succession throughout the perinatal period and its association with postpartum production traits in dairy cows: A review. Anim. Nutr..

[29] Chuang S.T., Ho S.T., Tu P.W., Li K.Y., Kuo Y.L., Shiu J.S., Wang S.Y., Chen M.J. (2020). The Rumen Specific Bacteriome in Dry Dairy Cows and Its Possible Relationship with Phenotypes. Animals.

[30] Xu S.Y., Feng X.R., Zhao W., Bi Y.L., Diao Q.Y., Tu Y. (2024). Rumen and hindgut microbiome regulate average daily gain of preweaning Holstein heifer calves in different ways. Microbiome.

[31] Jiang F., Gao Y., Peng Z., Ma X., You Y., Hu Z., He A., Liao Y. (2023). Isoacids supplementation improves growth performance and feed fiber digestibility associated with ruminal bacterial community in yaks. Front. Microbiol..

[32] Rabee A.E., Mohamed M.G.M., Sallam A., Elwakeel E.A., Mohammed R.S., Sabra E.A., Abdel-Wahed A.M., Mourad D.M., Hamed A.A., Hafez O.R. (2024). Rumen fermentation and microbiota in Shami goats fed on condensed tannins or herbal mixture. BMC Vet. Res..

[33] Martinez Boggio G., Monteiro H.F., Lima F.S., Figueiredo C.C., Bisinotto R.S., Santos J.E.P., Mion B., Schenkel F.S., Ribeiro E.S., Weigel K.A. (2024). Host and rumen microbiome contributions to feed efficiency traits in Holstein cows. J. Dairy Sci..

[34] Russell J.B., Rychlik J.L. (2001). Factors that alter rumen microbial ecology. Science.

[35] Plaizier J.C., Li S., Danscher A.M., Derakshani H., Andersen P.H., Khafipour E. (2017). Changes in Microbiota in Rumen Digesta and Feces Due to a Grain-Based Subacute Ruminal Acidosis (SARA) Challenge. Microb. Ecol..

[36] Elmhadi M.E., Ali D.K., Khogali M.K., Wang H. (2022). Subacute ruminal acidosis in dairy herds: Microbiological and nutritional causes, consequences, and prevention strategies. Anim. Nutr..

[37] Gorka P., Kowalski Z.M., Zabielski R., Guilloteau P. (2018). Invited review: Use of butyrate to promote gastrointestinal tract development in calves. J. Dairy Sci..

[38] Ma L., Yang Y., Liu W., Bu D. (2023). Sodium butyrate supplementation impacts the gastrointestinal bacteria of dairy calves before weaning. Appl. Microbiol. Biotechnol..

[39] Liu H., Han X., Zhao N., Hu L., Wang X., Luo C., Chen Y., Zhao X., Xu S. (2022). The Gut Microbiota Determines the High-Altitude Adaptability of Tibetan Wild Asses (Equus kiang) in Qinghai-Tibet Plateau. Front. Microbiol..

[40] Song Y., Sun L., Zhang S., Fan K., Wang H., Shi Y., Shen Y., Wang W., Zhang J., Han X. (2022). Enzymes and microorganisms jointly promote the fermentation of rapeseed cake. Front. Nutr..

[41] Jami E., White B.A., Mizrahi I. (2014). Potential role of the bovine rumen microbiome in modulating milk composition and feed efficiency. PLoS ONE.

[42] Jung J.S., Soundharrajan I., Kim D., Baik M., Ha S., Choi K.C. (2022). Microbiota and Serum Metabolic Profile Changes in Korean Native Hanwoo Steer in Response to Diet Feeding Systems. Int. J. Mol. Sci..

[43] Murray A.R. (2024). Supplementation Strategies for Growing and Finishing Beef Cattle on Tall Fescue Pastures in the Southeast.

